# How Times of Crisis Serve as a Catalyst for Creative Action: An Agentic Perspective

**DOI:** 10.3389/fpsyg.2020.600685

**Published:** 2021-01-07

**Authors:** Ronald A. Beghetto

**Affiliations:** Mary Lou Fulton Teachers College, Arizona State University, Tempe, AZ, United States

**Keywords:** creativity, COVID-19, uncertainty, agentic perspective, crisis

## Abstract

The human experience is punctuated by times of crisis. Some crises are experienced at a personal level (e.g., the diagnosis of a life-threatening disease), organizational level (e.g., a business facing bankruptcy), and still others are experienced on a societal or global level (e.g., COVID-19 pandemic). Although crises can be deeply troubling and anxiety provoking, they can also serve as an important catalyst for creative action and innovative outcomes. This is because during times of crisis our typical forms of reasoning and action may no longer serve us. It is precisely during such times that new ways of thought, action and leadership are needed. A key question for researchers to consider is: *Why and how times of crisis serve as an impetus for creative actions and outcomes?* The purpose of this paper is to address this question. I open by briefly discussing the features of a crisis. I then introduce an empirically testable, process model that outlines various pathways, factors, and outcomes associated with different ways people and organizations respond during times of crisis. I close by briefly outlining future directions for creativity theory and research.

## Introduction

The human experience is punctuated by times of crisis. Some crises are experienced at personal level (e.g., the diagnosis of a life-threatening disease), organizational level (e.g., a business facing bankruptcy), and still others are experienced on a societal or global level (e.g., COVID-19 pandemic). Although crises can be deeply troubling and anxiety provoking, they can also serve as an important catalyst for creative action and innovative outcomes at and beyond the individual level (Solnit, 2010; Cameron et al., 2018; Cohen and Cromwell, 2020; Leontidou, 2020). Moreover, these creative efforts can occur at individual, grassroots, and broader social levels. This is because during times of crisis our typical forms of reasoning and action may no longer serve us.

It is precisely during such times that new ways of thought, action and leadership are needed. Indeed, scholars have long recognized the creative potential of deep uncertainty. The early pragmatists, have for instance argued that it is only through states of doubt that we are compelled to think and act in new ways (Dewey, 1910; Peirce, 1958). Creativity researchers have similarly noted that it is from ill-defined problems that we are moved into creative and imaginative work (Getzels, 1964; Greene, 1995; Pretz et al., 2003; Craft, 2015).

A key question for creativity researchers to consider is: *How times of crisis serve as an impetus for creative actions and outcomes?* The purpose of this paper is to address this question. I open by briefly discussing the features of a crisis. I then introduce an empirically testable, process model that outlines various pathways, factors, and outcomes associated with different ways people and organizations respond during times of crisis. I close by briefly outlining future directions for creativity theory and research.

## A Time of Crisis: Defining Features

Prior to understanding how times of crisis can serve as a catalyst for creative and innovative outcomes, it is first important to understand the features of a crisis. The term *crisis* has its roots in the ancient Greek word *Kríno*, which means to judge or decide, and although crisis has come to mean different things in different disciplines it typically implies that decisive action is needed to avoid potentially negative consequences associated with the crisis situation. Taking action in times of crisis, however, is challenging because such experiences are shot through with uncertainty (Ansell and Boin, 2019; Beghetto, 2020; Cohen and Cromwell, 2020). We can thereby define a time of crisis *as an experience of profound uncertainty coupled with a sense of urgency to take action to minimize or avoid potential negative outcomes*. In considering the implications of this definition, it may be useful to further unpack some of its features.

The first feature pertains to an experience of uncertainty. Indeed, one of the markers of a crisis, is an experience of deep uncertainty (Ansell and Boin, 2019). Deep or profound uncertainties differ from other forms of uncertainty because they strike us as completely unknowable (Beghetto, 2020) and move us into a “state of genuine doubt” (Peirce, 1958, n.p.). It is under these states of genuine doubt that necessitate creative action, because former ways of thinking and acting are no longer viable. Moreover, in the info-digital age, where there is an abundance of information sources, the uncertainty people experience likely will be further compounded by conflicting and opposing perspectives rendering the situation as seemingly unknowable and thereby not actionable. It is not always problematic to leave profound uncertainties unresolved. When contemplating the profound uncertainties surrounding life’s greatest mysteries, a person may learn to live with the uncertainty. In times of crisis, however, leaving uncertainty unresolved is problematic because it is coupled with the second feature of a crisis: a sense of urgency to resolve it. Indeed, this sense of urgency is associated with a recognition that action is needed otherwise some negative consequences will follow. Heslop and Ormerod (2019) have discussed the unique sense of urgency experienced in a time of crisis, explaining that it falls somewhere in-between the more urgent experience of an *emergency* or *disaster* (which require immediate action) and the less urgent *risk* of a harmful situation, which occurs on a more “elongated timeframe” (p. 150) and thereby does not require immediate action.

In this way, a time of crisis results in a paradoxical experience of recognizing that somewhat urgent and decisive action is needed in a context of a seemingly unknowable and profoundly uncertain situation. Indeed, given that times of crisis present people and organizations with the double whammy of profound uncertainty and a sense of urgency to avoid future harm, it can result in people experiencing intense fear and anxiety. Indeed, as Grupe and Nitschke (2013) have reported, people experience states of heightened anxiety whenever they anticipate averse or harmful events *and* there is uncertainty surrounding those future events. A crisis carries both of these features and thereby can cause a range of responses, some of which can result in creative and innovative outcomes and others which can be considered maladaptive. How then might such a threatening and paradoxical situation result in people taking creative action?

## Potential Pathways Leading to Creative and Innovative Outcomes in Times of Crisis

**FIGURE 1:**
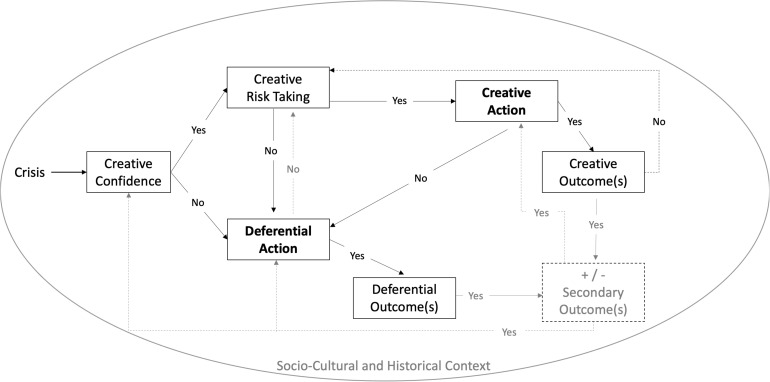
Agentic process model of creative actions and outcomes in times of crisis.

Figure 1 depicts a process model for considering different response pathways for people and organizations in time of crisis and potential outcomes associated with those pathways.

The model^1^ illustrated in Figure 1 represents an agentic perspective, which refers to actions and outcomes that are influenced by individual and organizational belief systems, prior behaviors, and environments, including more localized and broader socio-historical and cultural contexts (Bandura, 1997; Avery and Park, 2016; Karwowski and Beghetto, 2019). In this way, the model represents a focused perspective, one that conceptualizes creative action as intentional action and is inclusive of individual, organizational, and larger social actions and outcomes that start with the experience of a crisis and then diverges into different pathways and outcomes depending on a variety of factors.

The assertions offered by this model are general enough that researchers from various disciplines can use them as the basis for designing studies aimed at testing and refining them at different units of analysis (e.g., individual, organizational, and societal). This is not to say that there will not be important nuances and differences in how researchers apply these assertions in designing studies aimed at testing them at individual, organizational, and societal levels of analysis. Indeed, each level will require methodologies and measures tailored to the specific features of the study. Still, this framework offers a starting point for designing such studies, so that researchers can develop an understanding of how the assertions vary within and across different contexts and units of analyses.

The two different response pathways (creative vs. deferential action), can also be thought of as creativity and crisis-specific version of the classic approach| avoidance motivational orientation (Lewin, 1935), which refers to reasons why people literally or figuratively move toward (i.e., approach) taking positive actions vs. move away from (i.e., avoid) negative experiences or feared outcomes (Elliot, 2008). As will be discussed, a creative action (approach) orientation tends to be associated with creative outcomes; however, it is also possible for a blend of creative and deferential action (approach-avoid orientation) to result in creative outcomes (see also Roskes et al., 2012; Baas et al., 2020). Finally, the experience of crisis and the factors associated with the different pathways and outcomes are influenced by and influence the surrounding socio-cultural and historical context. With those features in mind, we can now turn our discussion to the different pathways, including the asserted factors and potential outcomes associated with them.

### Creative Confidence

**Assertion 1: Confidence in one’s ability to think and act creatively is a precursor to taking creative action during a crisis.**

This first assertion underscores the initial agentic component of creative action. Previous research has demonstrated that confidence plays an important role in moving from creative potential to creative action (Bandura, 1997; Karwowski and Beghetto, 2019; Puente-Diaz et al., 2019; Beghetto et al., 2020). This model asserts that confidence beliefs also play a key role in moving toward creative action under conditions marked by both heightened uncertainty and heightened awareness of an impending threat. Depending on the unit of analysis (individual, organizational, and societal) creative confidence can be conceptualized as an individual, collective belief and even some combination thereof (Goddard et al., 2004; Elliot et al., 2017).

Uncertainty, of course, disrupts people’s typical sense of control. However, unless people feel they have some ability to work through the uncertainty by thinking and acting creatively, then the stress and anxiety they experience likely will be compounded. Indeed, a perceived lack of control when experiencing a stressful event (i.e., a time of crisis), has been associated with depression, anxiety, and a sense of helplessness (Grupe and Nitschke, 2013). Consequently, the model asserts that unless people have some sense of agency via creative confidence, then it is likely that people would avoid creative action and move toward deferential action.

### Creative Risk Taking

**Assertion 2: Being willing to take creative risks is a necessary next step when moving toward and persisting in creative action.**

In addition to having creative confidence, people also need to be willing to take the risk of engaging in creative action. Indeed, recent research has demonstrated that the willingness to take risks plays an important moderating role in the link between creative confidence and creative action (Beghetto et al., 2020). Again, depending on the focus, willingness to take risks can be conceptualized as an individual, organizational, or even broader belief system.

During a time of crisis, creative risk taking may be viewed as more precarious given that perceived threats from the crisis are already salient. Still, in order for people to take creative action they will need to take some adaptive risks. This includes being able to make judgments about what risks are worth taking, what risks to avoid, and also discern when it may be more hazardous to not take a risk (Byrnes, 2011; Kaufman and Beghetto, 2013; Avery and Park, 2016; Jia et al., 2019). This model thereby asserts that although people may be more hesitant to take risks in a time of crisis, doing so is necessary to move toward creative action. Moreover, creative risk taking likely will also be required throughout later stages of the process, including in situations in which creative or deferential action does not lead to viable outcomes and thereby requires taking risks to creatively work through uncertainty and move toward the development of outcomes that help people navigate and address the potential hazards inherent in times of crisis.

### Creative Action

**Assertion 3: Creative action is an agentic response in times of crisis and, at a minimum, requires individual (organizational or societal) confidence to produce creative outcomes and a willingness to take the risks necessary to do so.**

Creative action represents an agentic response (Karwowski and Beghetto, 2019; Puente-Diaz et al., 2019) and refers to the willingness to think and act in new and different ways in an effort to navigate uncertainty and potential threats during times of crisis. Creative actions can range from more individual and localized efforts to organizational, societal, and even global endeavors. Individual people may, for instance, take creative actions on a more micro or personalized scale (e.g., individual people coming up with their own unique sanitizing procedures when bringing groceries and shipped items into their home; educators finding new ways to engage students through online instruction).

On a larger scale, organizations and groups may come up with new ways of working independently and together in an effort to respond creatively (e.g., restaurants pivoting their business models to leverage partnerships with local farmers to provide food preparation kits; professional sports organizations developing new protocols and procedures to offer sporting events; scientists working together around the globe to share data, findings, and insights in an effort to develop treatments). Regardless of the scale, however, taking creative action is no guarantee that doing so will result in creative or even beneficial outcomes. Indeed, as illustrated in the model and discussed below, even creative actions that lead to creative outcomes may have unintended secondary outcomes or even negative side-effects (Merton, 1936; Baert, 1991; Peterson and Skolits, 2019; Oliver et al., 2020). Another possible pathway, illustrated in the model, asserts that if creative action does not lead to what is judged to be a creative outcome *and* people are willing to continue to take creative risks of trying new things, then it may eventually result in creative outcomes. Otherwise, it likely will result in people deferring action (see differential action).

### Creative Outcomes

**Assertion 4: Creative activities during times of crisis can lead to outcomes that are judged to be new and meaningful, even if those outcomes are experienced on a more subjective level and temporary time scale.**

Although researchers and practitioners sometimes conceptualize creative action and creative outcomes as similar, the model highlights the importance of separating these two elements for research and practice. A person or group may, for instance, take creative action with the intention of making a positive contribution to oneself or others. However, the outcome of such actions can result in a variety of unintended and potentially harmful consequences (Merton, 1936; Baert, 1991; Moran, 2016). Indeed, the impact of creative action can be thought of as taking on a life of its own, which can be both beneficial (make a positive contribution) or problematic (result in harm). Consequently, distinguishing between actions and outcomes in the model can help researchers and practitioners evaluate and actively monitor the primary (and secondary) impact of seemingly well intended and beneficial creative action on themselves and others. Moreover, the primary outcomes can, over time and in different contexts, result in secondary outcomes, which also can range from potentially beneficial to potentially problematic. Specifying the difference between action and outcome highlights the need for conceptualizing and evaluating creative outcomes separately from creative actions, including the recognition that different people and groups may evaluate outcomes differently based on their unique contextual, socio-cultural, and historical vantage point (Baert, 1991; Peterson and Skolits, 2019; Oliver et al., 2020).

Indeed, creative outcomes that are produced during times of crisis can represent the full spectrum of creative contributions (Kaufman and Beghetto, 2009) — ranging from more subjective and intangible insights and experiences to more concrete outcomes that are recognized in and beyond the everyday environment (e.g., adoption of innovative treatment practices developed and shared amongst members of the international medical community). The model, however, posits that creative outcomes do not necessarily result in positive or lasting outcomes, even if the initial creativity of the ideas or activities was judged to be positive and viable. Creativity researchers have, for instance, recognized that creativity can and often does result in unintentional (and, in some cases, even intentional) negative outcomes (Baert, 1991; Cropley et al., 2010; Moran, 2016). The model thereby specifies that the creative outcomes that result from creative activity in times of crisis can run the gamut from causing personal and social harm (e.g., developing, selling, and using untested and potentially dangerous preventatives or treatments) to more positive and beneficial outcomes (e.g., scientists and medical professionals using appropriate protocols to develop and validate new or repurpose older approaches in an effort to help treat and prevent illness; teachers transforming safety screens on desks into cars windshields to make them more welcoming and less frightening for younger students to use). The model thereby takes creative outcomes one step further by highlighting that primary creative outcomes likely have secondary outcomes which may be positive, negative, or some combination thereof (See section “*secondary outcomes”*).

### Deferential Action

**Assertion 5: If people believe they have little to no agency during times of crisis, then they likely will defer their action to the guidance or direction of others.**

Deferential action refers here to people deferring to others in an effort to resolve the uncertainty they are facing and the actions they should take to avoid potential threats. Deferential action, similar to creative action, may result in beneficial outcomes, harmful outcomes, or some combination thereof. Depending on how intense the uncertainty is experienced and the level of perceived lack of agency, deferential action may take on more extreme forms. In the context of a crisis like COVID-19, for instance, this deferential action can include everything from denying that there is a crisis to feeling so helpless that people completely withdraw from taking any actions. These extremes represent the most problematic forms of deferential action and likely both come from an intense experience of uncertainty and perceived lack of agency.

Deferential action can also fall somewhere in between these extremes and reflect a blend of deferential conformity with some level of creative agency and action. Conforming to recommended safety guidelines (e.g., wearing protective face masks in public; organizations adhering to science-based, safety standards), while still engaging in some creative activities within those constraints (e.g., making one’s own face coverings; developing unique organizational protocols that enable organizations to safely function within recommended guidelines) would be an example of a more moderate and adaptive blend of a deferential-creative action approach. Indeed, conforming to health and safety directives also requires confidence in being able to do so. Avery and Park (2016), for instance, report on how crisis efficacy (i.e., people’s belief that they can conform to recommended behaviors during a crisis situation) is an important factor in following sensible health and safety directives during times uncertainty and crisis. It is therefore possible for human agency to still be exercised through an adaptive blend of deferential and adaptive creative actions.

A more problematic example of a blended approach would be to conform to fringe perspectives that are dismissive of the dangers of a given crisis, coupled with a choice to exercise one’s agency in defiance of individual and public safety guidelines and thereby increase the chances of harm for oneself and others. Crisis efficacy (Avery and Park, 2016), combined with creative metacognition (Bandura, 1997; Jia et al., 2019; Karwowski and Beghetto, 2019; Beghetto et al., 2020), likely plays an important role in whether people, organizations and societies can successfully navigate a blend of deferential and creative action in times of crisis and thereby maximize beneficial primary and secondary personal and social outcomes, while minimizing potential hazards.

### Deferential Outcomes

**Assertion 6: Deferential outcomes can range from positive to harmful, with the most beneficial being those that include a sensible blend of deferential action and creative action.**

The outcomes resulting from deferring action can include a variety of negative primary personal and social outcomes (Merton, 1936; Baert, 1991), including everything from dangerously unhealthy levels of stress, anxiety and depression (Grupe and Nitschke, 2013) to potentially negative social outcomes, such as social panic, unrest, and the development of beliefs that can lead to taking dangerous risks. In the case of a crisis like COVID-19, deferring to perspectives that deny or downplay the potential severity of the illness may lead to the further spread of the virus, protracting the duration of the crisis, and even causing serious illness and death (particularly to societies’ most vulnerable populations).

Deferential outcomes can, of course, also reflect positive primary benefits to individuals and larger societies, including helping to manage a crisis by conforming to the dynamic and evolving scientific, medical, and safety guidelines for how to respond in the given crisis situation. As discussed, the model asserts that beneficial deferential outcomes may be more likely to result from a blend of sensible deferential and creative action (i.e., engaging in new thoughts and actions in an effort to help navigate or resolve a crisis, while also actively working to identify and minimize potential hazards to oneself and others). Doing so still preserves a sense of agency (as opposed to a sense of helplessness), while still attempting to mitigate the potential hazards of a given crisis.

### Secondary Outcomes

**Assertion 7: Both creative and deferential outcomes will lead to a blend of positive and negative secondary outcomes.**

The model posits that secondary outcomes, which might be considered side-effects or unintended consequences, can be expected regardless of the pathway. As mentioned, creativity researchers have increasingly stressed the importance of recognizing that creative action and inaction can result in negative outcomes (Cropley et al., 2010) and therefore it is important to engage in creative actions from a more principled and ethical stance (Moran, 2016). This requires an ongoing effort to anticipate, monitor, identify, and address potentially harmful secondary outcomes (Peterson and Skolits, 2019; Oliver et al., 2020), which can emerge on an elongated time frame, when taking creative and deferential actions aimed at navigating and resolving the crisis.

## Future Directions for Creativity Theory and Research

The aim of this paper was to highlight how times of crisis can lead to creative or deferential actions and varied outcomes that can result from those actions, including the importance of monitoring immediate and long-term consequences of those actions (Baert, 1991; Moran, 2016; Oliver et al., 2020). I introduced an agentic process model that asserts different empirically testable pathways that can result from different responses in times of crisis. A key initial step in this work will be for researchers to identify existing and needed measures and methods, in and beyond the field of creativity studies, which can be adapted and developed to test and refine the assertions presented in this model across individual, organizational, and societal levels. Fortunately, there has been rapid and continuing growth in methodologies and measures, across various disciplines, that researchers can draw on when developing studies aimed at testing the model.

More specifically, researchers can adapt and build on existing methods, approaches and considerations for measuring individual and collective confidence beliefs (Goddard et al., 2004; Beghetto and Karwowski, 2017; Elliot et al., 2017; Karwowski and Kaufman, 2017; Puente-Diaz et al., 2019; Tang et al., 2020); creative risk-taking (e.g., Blais and Weber, 2006; Tyagi et al., 2017; Beghetto et al., 2020); creative activities and behaviors (Barbot et al., 2019; Plucker et al., 2019); deferential beliefs and behaviors (Barnett et al., 2014; Avery and Park, 2016); and methods for evaluations of primary and secondary outcomes (Argyris and Schön, 1974; Peterson and Skolits, 2019; Oliver et al., 2020). Depending on the particular aims and focus of such studies (e.g., testing the model at group vs. individual levels), researchers likely will need to modify and even develop new measures that are tailored to particular goals and contexts of their studies. Using more dynamic and micro-longitudinal methodologies (Barbot, 2019; Beghetto and Corazza, 2019), such as diary-based methods (Conner and Silvia, 2015; Bartlett and Milligan, 2020), and related experience sampling methods (Shiffman et al., 2008; Conner et al., 2009) seem particularly promising approaches for this work.

The model introduced in this paper serves a starting point. It is not meant to be exhaustive in specifying the full range of responses or outcomes available to individuals and groups when encountering a crisis. Indeed, the two featured responses, creative action and differential action offer only two (albeit broadly encompassing) possibilities. Future work can help clarify the elements of the model, including clarifying the areas of overlap, identifying boundaries between creative and deferential responses, and specifying other types of responses that may not be adequately accounted for by either a creative or deferential response. Moreover, the focus in this paper was on different responses in the midst of crisis, subsequent work should also examine the full temporal trajectory of creative and deferential responses and outcomes including prior to a crisis, during a crisis, and following a crisis (Baert, 1991; Avery and Park, 2016; Cameron et al., 2018; Cohen and Cromwell, 2020; Leontidou, 2020). This includes exploring the implications across the full temporal continuum at the individual, group, and broader societal level. Subsequent iterations of the model will also benefit from researchers working in interdisciplinary teams. Indeed, teams of interdisciplinary researchers drawing on existing literatures and approaches can go a long way in testing out, refining, and ultimately strengthening the model introduced in this paper.

I thereby invite researchers to develop this model by designing studies aimed at testing and refining the seven assertions associated with the pathways. Doing so has the potential to contribute to creativity theory and research and, more importantly, has the potential to contribute to our understanding of the role creativity might play in productively navigating uncertainty in times of crisis.

## Data Availability Statement

The original contributions presented in the study are included in the article/supplementary material, further inquiries can be directed to the corresponding author/s.

## Author Contributions

The author confirms being the sole contributor of this work and has approved it for publication.

## Conflict of Interest

The author declares that the research was conducted in the absence of any commercial or financial relationships that could be construed as a potential conflict of interest.

## Footnotes

1 Although the model introduced in this paper model is grounded in the creativity studies literature, it is important to note that there are numerous disciplines, literatures, methodologies, and practices beyond the field of creativity studies that can and should be drawn on to inform, further develop, and strengthen the applicability of this model and the elements represented in the model (e.g., political philosophy, counseling psychology, STEM psychology, sociocultural psychology, organizational psychology, and sociology just to name a few). I would like to thank one of the reviewers for stressing this point and I have attempted to incorporate a few relevant examples as suggested by that reviewer throughout. The model offered here is intended to be a starting point and will only benefit from including a more diverse range of interdisciplinary perspectives, concepts, and ideas and practices within and across fields of study.
